# Iron Corrosion via Direct Metal-Microbe Electron Transfer

**DOI:** 10.1128/mBio.00303-19

**Published:** 2019-05-14

**Authors:** Hai-Yan Tang, Dawn E. Holmes, Toshiyuki Ueki, Paola A. Palacios, Derek R. Lovley

**Affiliations:** a Department of Microbiology, Morrill IV Science Center, University of Massachusetts Amherst, Amherst, Massachusetts, USA; b Jiangsu Provincial Key Lab for Organic Solid Waste Utilization, National Engineering Research Center for Organic-based Fertilizers, Jiangsu Collaborative Innovation Center for Solid Organic Waster Resource Utilization, Nanjing Agricultural University, Nanjing, China; c Department of Physical and Biological Sciences, Western New England University, Springfield, Massachusetts, USA; d Department of Biology, University of Southern Denmark, Odense, Denmark; University of Delaware

**Keywords:** *Geobacter*, autotroph, cytochrome, electromicrobiology, extracellular electron transfer, zero-valent iron

## Abstract

The concept that anaerobic microorganisms can directly accept electrons from Fe(0) has been controversial because direct metal-microbe electron transfer has previously only been indirectly inferred. Fe(0) oxidation was studied with Geobacter sulfurreducens strain ACL, an autotrophic strain that was previously shown to grow with electrons derived from a graphite cathode as the sole electron donor. Strain ACL grew with Fe(0) as the sole electron donor and fumarate as the electron acceptor. However, it appeared that at least a portion of the electron transfer was via H_2_ produced nonenzymatically from the oxidation of Fe(0) to Fe(II). H_2_, which accumulated in abiotic controls, was consumed during the growth of strain ACL, the cells were predominately planktonic, and genes for the uptake hydrogenase were highly expressed. Strain ACL_HF_ was constructed to prevent growth on H_2_ or formate by deleting the genes for the uptake of hydrogenase and formate dehydrogenases from strain ACL. Strain ACL_HF_ also grew with Fe(0) as the sole electron donor, but H_2_ accumulated in the culture, and cells heavily colonized Fe(0) surfaces with no visible planktonic growth. Transcriptomics suggested that the outer surface *c*-type cytochromes OmcS and OmcZ were important during growth of strain ACL_HF_ on Fe(0). Strain ACL_HF_ did not grow on Fe(0) if the gene for either of these cytochromes was deleted. The specific attachment of strain ACL_HF_ to Fe(0), coupled with requirements for known extracellular electrical contacts, suggest that direct metal-microbe electron transfer is the most likely option for Fe(0) serving as an electron donor.

## Abstract (precis)

The anaerobic corrosion of iron structures is expensive to repair and can be a safety and environmental concern. It has been known for over 100 years that the presence of anaerobic respiratory microorganisms can accelerate iron corrosion. Multiple studies have suggested that there are sulfate reducers, methanogens, and acetogens that can directly accept electrons from Fe(0) to support sulfate or carbon dioxide reduction. However, all of the strains studied can also use H_2_ as an electron donor for growth, which is known to be abiotically produced from Fe(0). Furthermore, no proteins definitely shown to function as extracellular electrical contacts with Fe(0) were identified. The studies described here demonstrate that direct electron transfer from Fe(0) can support anaerobic respiration. They also map out a simple genetic approach to the study of iron corrosion mechanisms in other microorganisms. A better understanding of how microorganisms promote iron corrosion is expected to lead to the development of strategies that can help reduce adverse impacts from this process.

## INTRODUCTION

The mechanisms by which microorganisms accelerate the corrosion of iron are of interest because of the costs and threats to the environment and human safety associated with the corrosion of steel (1–3). Iron corrodes when metallic iron [Fe(0)] is oxidized to Fe(II):

(1) $$\text{Fe}\left(0\right)\to \text{Fe}\left(\text{II}\right)+{\text{2e}}^{-}$$

This oxidation reaction must be coupled with a corresponding reduction reaction, which under anaerobic conditions is typically the reduction of protons to produce H_2_:

(2) $${\text{2H}}^{+}{\text{+2e}}^{-}\to H_{2}$$

With the net reaction:

(3) $$\text{Fe}\left(0\right)+{\text{2H}}^{+}\to \text{Fe}\left(\text{II}\right)+H_{2}$$

Microbial removal of H_2_ theoretically favors additional H_2_ formation and iron corrosion (3). Thus, H_2_-consuming microorganisms, such as sulfate-reducing (equation 4), methane-producing (equation 5), and acetogenic bacteria (equation 6), may have an important influence on corrosion (4–6).

(4) $${\text{4H}}_{\text{2}}+{\text{SO}}_{\text{4}}{}^{\text{2}-}+{\text{H}}^{+}\to {\text{HS}}^{-}+{\text{4H}}_{\text{2}}\text{O}$$

(5) $${\text{4H}}_{\text{2}}+{\text{HCO}}_{\text{3}}{}^{-}+{\text{H}}^{+}\to {\text{CH}}_{\text{4}}+{\text{3H}}_{\text{2}}\text{O}$$

(6) $${\text{4H}}_{\text{2}}+{\text{2HCO}}_{\text{3}}{}^{-}+{\text{H}}^{+}\to {\text{CH}}_{\text{3}}{\text{COO}}^{-}+{\text{4H}}_{\text{2}}\text{O}$$

Removal of Fe(II), the other product of Fe(0) oxidation, also favors Fe(0) oxidation. Microbial production of sulfide during sulfate reduction provides an Fe(II) sink:

(7) $$H_{2}\text{S}+\text{Fe}\left(0\right)\to \text{FeS}+H_{2}$$

Thus, investigations into the mechanisms of corrosion in the presence of sulfate reducers must account for this influence on corrosion rates.

A number of studies have suggested that the most important microbial contribution to corrosion is direct metal-microbe electron transfer, in which the electrons derived from Fe(0), rather than a H_2_ intermediate, serve as the electron donor for anaerobic respiration (1, 2, 4–9). It is important to recognize that there was no direct demonstration of direct electron transfer in any of these studies. All of the microbes proposed to directly accept electrons from Fe(0) could use H_2_ as an electron donor, and the possibility of H_2_ serving as an electron carrier during Fe(0) oxidation was not ruled out. Rather, the claim for direct electron transfer was based on the observation that the microbes that were proposed to be capable of direct electron transfer oxidized Fe(0) more quickly than did other closely related H_2_-utilizing strains.

However, the enrichment of the microbes proposed to be capable of direct electron transfer on Fe(0) may have selected for other characteristics that promote Fe(0) oxidation with the production of H_2_ (10). For example, many of the microbes proposed to be capable of direct electron transfer from Fe(0) appear to be more effective in colonizing surfaces, which may result in more effective H_2_ removal at the point of production. Another consideration is that the slow release of H_2_ from Fe(0) oxidation may enrich for strains with higher affinities for H_2_, thereby enhancing H_2_ uptake at the Fe(0) surface compared with closely related strains that were enriched and isolated with high concentrations of H_2_.

Studies with microbial strains incapable of using H_2_ as an electron donor for growth have more definitively demonstrated direct electron transfer from other types of insoluble electron donors. Direct electron transfer from graphite electrodes to support anaerobic respiration was demonstrated with Geobacter metallireducens, which is unable to respire H_2_ (11, 12), and a strain of G. sulfurreducens in which the gene for the uptake hydrogenase (13) was deleted (14). In a similar manner, construction of a strain that eliminated the possibility that H_2_ or formate could function as an electron donor for G. sulfurreducens clarified its ability to participate as the electron-accepting partner in direct interspecies electron transfer (15, 16).

Multiheme outer surface *c*-type cytochromes are key electrical contacts between Geobacter species and other species, electrodes, and minerals (17–19). Some microbes proposed to be capable of directly accepting electrons from Fe(0) have multiheme outer surface *c*-type cytochromes (20, 21). However, the ability of cytochromes to function as the direct electrical contacts on the outer surface has not been demonstrated.

The recent construction of a strain of G. sulfurreducens capable of autotrophic growth (22) has provided the opportunity to further explore the possibility of direct electron transfer from Fe(0) in a genetically tractable microbe known to be highly effective in extracellular electron exchange. Here, we report evidence for direct electron transfer from Fe(0) under conditions in which the possibility of H_2_ (or formate) serving as an intermediate electron carrier has been eliminated, and we identify likely multiheme *c*-type cytochrome electrical contacts with Fe(0).

## RESULTS AND DISCUSSION

### H_2_ as an electron carrier during growth of strain ACL with Fe(0) as the electron donor.

**FIG 1:**
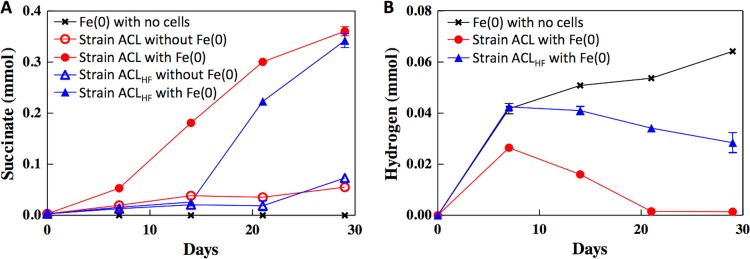
(A and B) Succinate (A) and hydrogen (B) concentrations over time when strain ACL and strain ACL_HF_ were grown with and without Fe(0) as the potential electron donor and fumarate as the electron acceptor. Error bars represent one standard deviation of the mean of the results from triplicate cultures.

G. sulfurreducens strain ACL grew in medium with Fe(0) as the sole electron donor with the reduction of fumarate to succinate (Fig. 1). The culture was sequentially transferred (5% inoculum) with similar rates of succinate production. No succinate was produced in the controls of cells without Fe(0) or medium with Fe(0) but no cells (Fig. 1A).

H_2_ accumulated in the presence of Fe(0) when cells were not added (Fig. 1B), consistent with the well-known abiotic corrosion of Fe(0) (23). There was no H_2_ production in the absence of Fe(0). In the presence of cells of strain ACL and Fe(0), H_2_ accumulated and then declined, indicating that H_2_ was being consumed in the presence of strain ACL.

**FIG 2:**
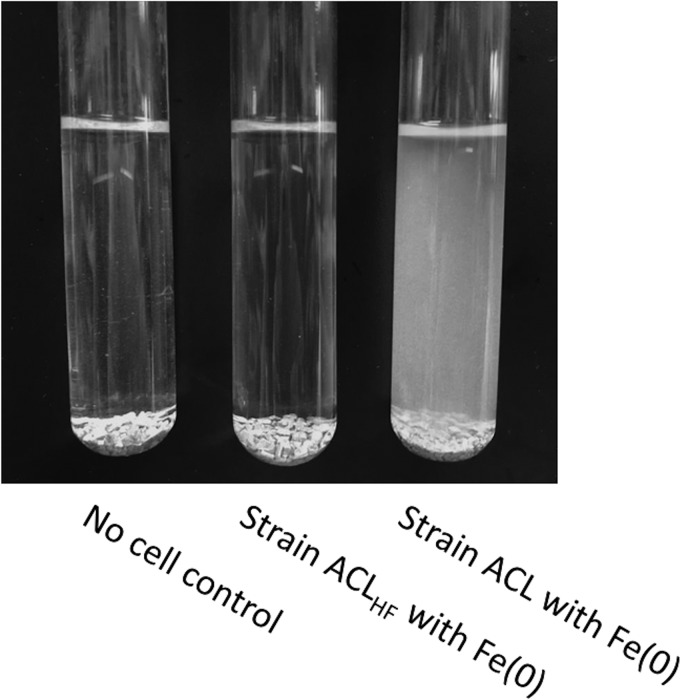
Appearance of cultures after growth of strain ACL or strain ACL_HF_ in medium with Fe(0) provided as the electron donor and fumarate provided as the electron acceptor.

**FIG 3:**
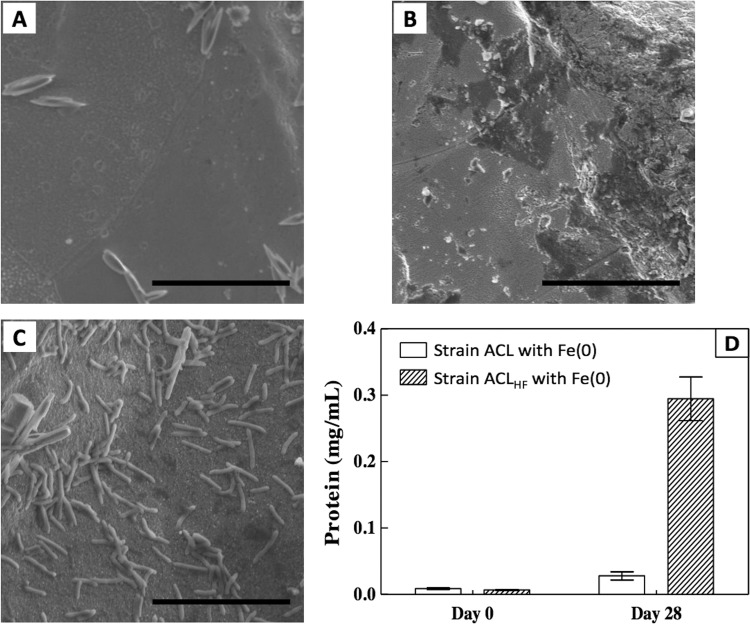
Scanning electron micrograph images of Fe(0) particles. (A) No-cell control. (B) Strain ACL after 28 days of incubation. (C) Strain ACL_HF_ after 28 days of incubation. Scale bar = 10 μm. (D) Protein concentrations detected on Fe(0) particles. Error bars represent one standard deviation of the mean of the results from triplicate samples.

Cultures of strain ACL growing on Fe(0) were visibly turbid (Fig. 2), and few cells were associated with the Fe(0) particles (Fig. 3B and D). These results suggested that strain ACL was primarily growing with H_2_ as the electron donor because cells do not need to attach to the Fe(0) particle surface to metabolize H_2_. This is similar to previous studies in which G. sulfurreducens did not attach during syntrophic growth with a H_2_-producing partner (16). In contrast, G. sulfurreducens grows in aggregates with an electron-donating partner when electrons are delivered via direct interspecies electron transfer (15, 16, 24).

### Direct electron transfer from Fe(0) with strain ACL_HF_.

Previous studies have demonstrated that deletion of the gene for the uptake hydrogenase (*hybL*) and the formate dehydrogenase (*fdnG*) yielded a strain of G. sulfurreducens that could not grow with H_2_ or formate as the electron donor (16). Therefore, in order to better evaluate the possibility of direct electron transfer from Fe(0), the previously described (16) G. sulfurreducens Δ*fdnG* Δ*hybL* mutant strain unable to use H_2_ or formate (16) was modified with the insertion of the *aclA* and *aclB* genes for citrate lyase in the same manner previously described (22) to construct strain ACL (Fig. S1). This new strain was designated strain ACL_HF_.

10.1128/mBio.00303-19.1 FIG S1 The gene clusters for formate dehydrogenase (*fdnG*, *fdnH*, *fdnI*, *fdhD*, and *fdnT*) (GSU0777 to GSU0781) and Hyb hydrogenase (*hybS*, *hybA*, *hybB*, *hybL*, *hybP*, and *hybT*) (GSU0782 to GSU0787) are indicated in green and blue, respectively. Disruption of *fdnG* and *hybL* is indicated by X. *aclA* and *aclB*, citrate lyase; *lacI*, Lac repressor; P/O, *tac-lac* promoter/*lac* operator; *apr^r^*, apramycin resistance; *gltA*, citrate synthase (GSU1106); *ammecr1*, AMMECR1 family protein (GSU1107); *hp*, hypothetical protein (GSU3507). Download FIG S1, TIF file, 1.5 MB. Copyright © 2019 Tang et al. 2019 Tang et al. This content is distributed under the terms of the Creative Commons Attribution 4.0 International license.

When strain ACL_HF_ was inoculated into medium with Fe(0) as the electron donor and fumarate as the electron acceptor, succinate production lagged initially but once initiated was more rapid than succinate production by strain ACL (Fig. 1A). Unlike strain ACL, strain ACL_HF_ did not consume substantial H_2_ during the reduction of fumarate to succinate (Fig. 1B).

In contrast to the turbid strain ACL cultures, cultures of strain ACL_HF_ lacked turbidity despite comparable succinate production (Fig. 2). Scanning electron microscopy revealed that over time, strain ACL_HF_ heavily colonized the Fe(0) particles (Fig. 3C), and protein concentrations on the Fe(0) surface also increased over time (Fig. 3D). Attachment is required for direct electron transfer (18). These results suggest that strain ACL_HF_ adapted to the inability to use H_2_ as an electron donor by attaching to Fe(0) for Fe(0) oxidation.

### Transcriptomic and genetic analyses of direct electron transfer mechanisms.

In order to gain further insight into the potential mechanisms for electron transfer from Fe(0), the transcriptomes of strain ACL and strain ACL_HF_ grown on Fe(0) were analyzed (Table S1). In strain ACL, log_2_ reads per kilobase per million (RPKM) values for genes associated with the uptake hydrogenase complex (*hybS, hybA, hybB, hybL, hybP,* and *hybT*) ranged from 4.4 ± 0.2 to 8.1 ± 0.1, which was substantially higher than the median log_2_ RPKM value for genome-wide expression of strain ACL (2.5 ± 0.1). In contrast, log_2_ RPKM values for genes coding for formate dehydrogenase (*fdnG, fdnH,* and *fdnI*) ranged from 1.03 ± 0.5 to 2.03 ± 0.5 and were well below the median log_2_ RPKM. These results further suggest that H_2_ was an important intermediary carrier for electron transfer from Fe(0) to strain ACL but that formate was not.

10.1128/mBio.00303-19.2 TABLE S1 (A) Genes that were being transcribed at levels above the median log_2_ RPKM value in ACL cells grown with zero-valent iron (ZVI) and hydrogen as the electron donor and fumarate as the electron acceptor. (B) Genes that were being transcribed at levels above the median log_2_ RPKM value in ACL_HF_ cells grown with ZVI as the electron donor and fumarate as the electron acceptor. Download Table S1, XLSX file, 0.2 MB. Copyright © 2019 Tang et al. 2019 Tang et al. This content is distributed under the terms of the Creative Commons Attribution 4.0 International license.

The median log_2_ RPKM value for the ACL_HF_ cells (1.3 ± 0.6) was substantially lower than the median log_2_ RPKM value for ACL cells (2.5 ± 0.1), yet the transcript abundances for the genes for the multiheme *c*-type cytochromes OmcS and OmcT were more than 4-fold higher in strain ACL_HF_ than in strain ACL (Table 1). *omcS* and *omcT* are adjacent on the G. sulfurreducens chromosome (25). *omcT* is cotranscribed with *omcS*, but *omcS* can also be transcribed separately (25). OmcS is one of the most abundant outer surface proteins during growth on Fe(III) and Mn(IV) oxides (25–27); *omcS* is highly expressed when G. sulfurreducens functions as the electron-accepting partner for direct interspecies electron transfer in coculture with *G. metallireducens* (15), and OmcS is important for electron transfer to anodes under some conditions (28). In contrast, OmcT is in low abundance under all growth conditions that have been evaluated (25, 27). None of the other genes with higher expression in the ACL_HF_ strain had annotations that suggested that they could have a direct function in electron transfer from Fe(0) (Table 1).

**TABLE 1:** Ten most highly upregulated genes in ACL_HF_ strain compared to the ACL strain^*a*^

Name	Annotation	Abbreviation	Main role	Specific role	ACL_HF_ log_2_ RPKM	ACL log_2_ RPKM	Fold upregulated in ACL_HF_
GSU3506	DUF2917 domain protein		Unknown function		3.99	0.62	17.22
GSU0012	Protoporphyrinogen oxidase	*hemG*	Biosynthesis of cofactors, prosthetic groups, and carriers	Heme, porphyrin, and cobalamin	4.95	2.41	7.67
GSU0769	Protein RarD	*rarD*	Transport and binding proteins	Unknown substrate	3.08	1.11	5.96
GSU0013	Transcriptional regulator, MarR family	*marR*	Regulatory functions	DNA interactions	4.20	2.40	5.84
GSU2503	*c*-type cytochrome	*omcT*	Energy metabolism	Electron transport	4.50	2.06	5.32
GSU2504	*c*-type cytochrome	*omcS*	Energy metabolism	Electron transport	4.97	2.80	4.45
GSU0018	Transcriptional regulator, GntR family	*gntR*	Regulatory functions	DNA interactions	3.77	2.26	4.22
GSU3410	Putative membrane protein		Unknown function		8.35	6.83	3.11
GSU3395	l-Proline dehydrogenase	*putA*	Energy metabolism	Amino acids and amines	2.93	1.99	2.86
GSU3409	Putative membrane protein		Unknown function		5.07	3.84	2.64

a Genes that were significantly upregulated in the ACL_HF_ strain compared to the ACL strain when grown on Fe(0) with fumarate as the electron acceptor. The median log_2_ RPKM value for the ACL_HF_ strain was 1.53, and the median log_2_ RPKM value for the ACL strain was 3.31.

**FIG 4:**
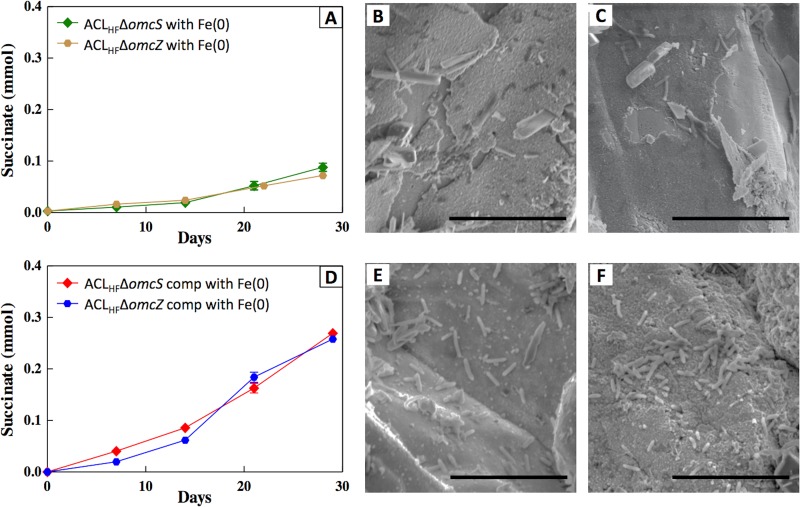
Impact of deleting the gene for the outer surface *c*-type cytochrome OmcS or OmcZ. (A) Succinate production from fumarate reduction in strains with either *omcS* or *omcZ* deleted. (B and C) Scanning electron micrographs demonstrating the lack of cell growth on Fe(0) in the *omcS* (B) or *omcZ* (C) mutants. (D) Succinate production from fumarate reduction when the *omcS* or *omcZ* deletion mutants were complemented in *trans*. (E and F) Growth of cells on Fe(0) when the Δ*omcS* mutants (E) or Δ*omcZ* mutants (F) were complemented in *trans*. Error bars represent one standard deviation from the mean of the results from triplicate cultures. Scale bar = 10 μm.

When *omcS* was deleted, the mutant strain failed to grow on Fe(0) (Fig. 4). The capacity for growth on Fe(0) was restored when *omcS* expression was complemented in *trans* (Fig. 4). These results suggested that OmcS is an important component in electron transport from Fe(0). Under some conditions, the multiheme *c*-type cytochrome OmcZ, rather than OmcS, is an important electrical contact for electron transfer to electrodes (29, 30). The log_2_ RPKM values for *omcZ* transcripts in strain ACL (5.1 ± 0.1) and strain ACL_HF_ (4.5 ± 0.6) were comparable. Considering the much lower median log_2_ RPKM value for strain ACL_HF_, this result indicated a higher relative expression of *omcZ* in strain ACL_HF_. Deletion of the gene for *omcZ* prevented growth on Fe(0) (Fig. 4). Growth was restored with in *trans* complementation of *omcZ*. These results suggest that OmcZ is also involved in electron transfer from Fe(0) into the cell.

Immunogold labeling has demonstrated that both OmcS (26, 31) and OmcZ (32) can associate with the outer cell surface and thus are properly localized to function as electrical contacts between cells and Fe(0). Under some conditions, OmcS may also extend at distance from the cell, either attached to pili (26, 33, 34) or as filaments composed of OmcS (35). However, filament extensions are unlikely to be important for electron transfer from Fe(0) because the cells are in close contact with the Fe(0) surface.

The inability of strain ACL_HF_ to grow on Fe(0) in the absence of *omcS* or *omcZ* contrasts with the previous finding that the deletion of *omcS* or *omcZ* did not inhibit electron uptake of wild-type G. sulfurreducens from negatively poised graphite cathodes (36). However, there are substantial differences between the two studies. In the cathode study, electron uptake, not growth, in a pregrown biofilm was monitored. Different electron transport pathways into the cell may be required to generate sufficient ATP to support growth. The graphite cathodes and Fe(0) have very different surface properties, including a much lower potential for the Fe(0)/Fe(II) redox couple (−470 mV standard hydrogen electrode [SHE] [1]) than the electrode poise (−300 mV SHE [36]). Furthermore, the previous studies on electron uptake from cathodes (36) were conducted with wild-type G. sulfurreducens, which required acetate in the medium as a carbon source. As previously discussed in detail (22), the added acetate may have also served as an electron donor, resulting in different routes for electron flux in the wild-type strain from those found in the autotrophic strain, which was grown in the absence of acetate.

### Implications.

To our knowledge, strain ACL_HF_ is the first microorganism for which it can reliably be concluded that Fe(0) functions as a direct electron donor. Unlike previous studies, the possibility of H_2_ or formate serving as an electron carrier was eliminated, and the need for outer surface redox-active electrical contacts was confirmed. Although it was suggested that a nitrate-reducing strain of Prolixibacter that could not grow with H_2_ as the sole electron donor might be capable of directly accepting electrons from Fe(0) (37), H_2_ was consumed in Fe(0)-amended cultures, indicating that there was a mechanism for H_2_ uptake. In contrast, strain ACL_HF_ did not consume the H_2_ produced from abiotic Fe(0) oxidation.

Strain ACL_HF_ can serve as a model strain for developing a better understanding of direct electron transfer from Fe(0) and its potential role in corrosion. These studies can build on previous studies regarding extracellular electron exchange in G. sulfurreducens (38–41) to develop testable hypotheses regarding the mechanisms for electron transfer from Fe(0). Studies with strain ACL_HF_ also have the advantage that, unlike studies with sulfate reducers, the production of sulfide, which can nonenzymatically promote Fe(0) oxidation (1), is avoided. Growth of strain ACL_HF_ on Fe(0) also does not result in the formation of organic acids, which can promote corrosion (42, 43).

Furthermore, the finding that direct electron transfer from Fe(0) is possible provides further incentive for developing genetic approaches to better evaluate whether the direct electron transfer that has been proposed for other respiratory classes of microorganisms is feasible. Constructing strains that are unable to use H_2_ or formate as an electron donor is essential for definitive studies. For example, Fe(0) is rapidly oxidized in the presence of Desulfovibrio ferrophilus strain IS5, which is proposed to directly accept electrons from Fe(0) (1). Adapting strategies that are well developed for the genetic manipulation of other Desulfovibrio species (44–46), coupled with biochemical localization of putative electrical contacts, would more rigorously test the hypothesis of direct electron transfer from Fe(0) for this microbe, as well as potential alternative strategies, such as a role for flavin shuttles (47). Similar approaches applied to diverse microorganisms are likely to identify unifying mechanisms for direct electron transfer from Fe(0) that will greatly aid in elucidating the capacity for direct metal-microbe electron transfer (DMMET) in the microbial world and may lead to strategies to mitigate corrosion.

## MATERIALS AND METHODS

### Bacterial strains and growth condition.

All strains were routinely maintained under strict anaerobic conditions at 30°C in NB medium (per liter of medium: 10 ml 100X NB salts, 10 ml 100X NB mineral elixir, 15 ml DL vitamins, 0.04 g CaCl2·2H20, 0.1 g MgSO4·7H20, 1.8 g NaHCO3, 0.5 g Na2CO3·H20, 1 μM Na2SeO4) in which fumarate (40 mM) was supplied as the electron acceptor and acetate (15 mM) was the electron donor (48). For growth on Fe(0), the acetate was omitted, and zero-valent iron granules (1 to 2 mm in diameter; Alfa Aesar, Inc.) were provided as the electron donor in 10 ml of medium dispensed into 28-ml anaerobic pressure tubes under an N_2_:CO_2_ [80:20 (vol/vol)] atmosphere.

Geobacter sulfurreducens strain ACL, which is capable of autotrophic growth due to the introduction of the citrate lyase genes *aclA* and *aclB* (22), was obtained from our laboratory culture collection. Strain ACL_HF_ was constructed by using the previously described (22) procedure to insert these same citrate lyase genes into the chromosome of a previously described (16) G. sulfurreducens strain in which the genes for the uptake hydrogenase (*hybL*) and formate dehydrogenase (*fdnG*) were deleted.

Additional strains were constructed in the strain ACL_HF_ background in which the gene for OmcS or the gene for OmcZ was deleted. The sequences of all primer pairs used for the construction of deletion mutant strains and their complements are listed in Table S2. All mutants were constructed by replacement of the gene of interest with a gentamicin resistance cassette, as previously described (49).

10.1128/mBio.00303-19.3 TABLE S2 Primers used to construct various deletion mutants and complement strains. Download Table S2, DOCX file, 0.02 MB. Copyright © 2019 Tang et al. 2019 Tang et al. This content is distributed under the terms of the Creative Commons Attribution 4.0 International license.

Primer pairs were designed to amplify regions approximately 500 bp upstream and downstream of the target genes and to add AvrII (CCTAGG) restriction sites to the PCR products. These products were ligated into the pCR2.1 TOPO cloning vector, resulting in the formation of PCR2.1up5′+3′dn. Plasmids carrying *omcS* and omcZ mutant alleles were linearized by digestion with KpnI (GGTACC) and SacI (GAGCTC), respectively. Linearized plasmids were transformed into ACL_HF_ competent cells by electroporation, as previously described (48), and resulted in the formation of ACL_HF_ Δ*omcS* and ACL_HF_ Δ*omcZ* strains. Isolated colonies were grown on agar plates supplemented with fumarate (50 mM), acetate (10 mM), and gentamicin (20 μg/ml).

Deletion mutants made in this study were complemented by transformation with recombinant plasmids carrying a constitutive *lac* promoter (pCM66) and the *omcS* or *omcZ* gene with their native ribosome binding sites (50). Primers (Table S2) were designed to introduce XbaI (TCTAGA) and BamHI (GGATCC) restriction sites into the *omcS* amplicon and EcoRI (GAATTC) and HindIII (AAGCTT) restriction sites into the *omcZ* amplicon. After the complementary plasmids were constructed, they were introduced into ACL_HF_ Δ*omcS* and ACL_HF_ Δ*omcZ* mutant cells by electroporation, as previously described (48).

### Transcriptomics.

Cells were harvested from triplicate 50-ml cultures of strain ACL_HF_ and strain ACL grown as described above with Fe(0) as the potential electron donor and fumarate as the acceptor. Cultures were mixed with RNAprotect (Qiagen) in a 1:1 ratio, and iron particles and cells were pelleted by centrifugation at 3,000 × *g* for 15 min at 4°C, as previously described (22). Pellets were then immediately frozen in liquid nitrogen and stored at −80°C. Total RNA was extracted from all six Fe(0)-containing cell pellets according to the previously described protocol (51) and cleaned with the RNeasy minikit (Qiagen). All RNA samples were then treated with Turbo DNA-free DNase (Ambion, Austin, TX). In order to ensure that samples were not contaminated with genomic DNA, PCR with primers targeting the 16S rRNA gene was done with RNA that had not been reverse transcribed. Further enrichment of mRNA was done with the MICROB*Express* kit (Ambion), according to the manufacturer’s instructions.

Directional multiplex libraries were prepared with the ScriptSeq v2 RNA sequencing (RNA-seq) library preparation kit (Epicentre), and paired-end sequencing was performed with a HiSeq 2000 platform at the Deep Sequencing Core Facility at the University of Massachusetts Medical School in Worchester, MA.

All raw data generated by Illumina sequencing were quality checked by visualization of base quality scores and nucleotide distributions with FastQC (http://www.bioinformatics.babraham.ac.uk/projects/fastqc/). Initial raw nonfiltered forward and reverse sequencing libraries contained an average of 52,114,626 ± 10,712,759 reads that were ∼100 bp long. Sequences from all of the libraries were trimmed and filtered with Trimmomatic (52) with the sliding window approach set to trim bases with quality scores lower than 3, strings of 3+ Ns, and reads with a mean quality score lower than 20. Bases were also cut from the start and end of reads that fell below a threshold quality of 3, and any reads smaller than 100 bp were eliminated from the library. These parameters yielded an average of 47,913,813 ± 14,703,090 trimmed quality reads per RNA-seq library.

All paired-end reads were then merged with FLASH (53), resulting in 19,462,441 ± 9,267,108 reads, with an average read length of 147 ± 45 bp. After merging the quality control (QC)-filtered reads, SortMeRNA (54) was used to separate all rRNA reads from nonribosomal reads.

Trimmed and filtered mRNA reads from the triplicate samples for the three different culture conditions were mapped against the Geobacter sulfurreducens strain PCA genome (NCBI RefSeq accession no. NC_002939.5) downloaded from GenBank at the National Center for Biotechnology Information (NCBI) website (https://www.ncbi.nlm.nih.gov). Mapped reads were normalized with the reads per kilobase per million (RPKM) mapped reads method (55, 56) using the ArrayStar software (DNAStar). Analysis of reads from all three biological replicates for each condition demonstrated that the results were highly reproducible. Therefore, all reported values were obtained after merging and averaging replicates. Expression levels were considered significant only when the log_2_ RPKM value was higher than that of the median log_2_ RPKM.

### Analytical methods.

The formation of succinate was monitored over time with Shimadzu high-performance liquid chromatography (HPLC) with an Aminex HPX-87H ion exclusion column (300 mm by 7.8 mm) and an eluent of 8.0 mM sulfuric acid, as previously described (57).

Hydrogen was measured from the headspace of cultures at regular intervals using strictly anaerobic sampling techniques. Headspace gas was monitored with a gas chromatograph (GC; Agilent Technologies G1530A, USA) equipped with a Carboxen-1010 Plot column (30 m by 0.53 mm) and a thermal conductivity detector. The oven temperature was 40°C, and the detector temperature was set at 225°C. The carrier gas was N_2_.

For protein extraction from Fe(0) particles, 10-ml cultures were centrifuged at 3,000 × *g* for 20 min at 4°C, and 2 ml of 5% SDS solution was added to the pelleted particles. Pellets were then steam treated for 15 min and centrifuged at 16,100 × *g* for 20 min at 4°C. Supernatant was collected, and protein concentrations were determined using the Pierce bicinchoninic acid (BCA) protein assay kit (Thermo Fisher Scientific, USA), according to the manufacturer’s instructions.

### Scanning electron microscopy.

Fe(0) particles were collected from cultures when succinate production plateaued and were fixed with 2.5% glutaraldehyde in 0.1 M phosphate buffer for 12 h at 4°C. They were then washed 3 times in 0.1 M phosphate buffer at 4°C for 10 min and then dehydrated in successive ethanol-water mixtures of 35%, 50%, 70%, 80%, 90%, 95%, and 100% for 10 min. The 100% ethanol step was repeated 3 times. Samples were further dehydrated in a 50% hexamethyldisilazane (Sigma-Aldrich, St. Louis, MO, USA) ethanol solution by gentle mixing for 3 min at room temperature, immersed in pure hexamethyldisilazane for 3 min at room temperature, and dried with a stream of high-purity nitrogen for 30 min. Scanning electron microscopy was conducted with an ultrahigh-resolution field emission scanning electron microscope (FEI Magellan 400; Nanolab Technologies, CA, USA).

### Data availability.

Illumina sequence reads have been submitted to the NCBI database under BioProject number PRJNA510956 and submission number SUB4929798.
