# Supplementary figures and images for: Iron Corrosion via Direct Metal-Microbe Electron Transfer
Source: mBio. 2019 May 14;10(3):e00303-19. doi: 10.1128/mBio.00303-19 (PMC6520446; doi:10.1128/mBio.00303-19)

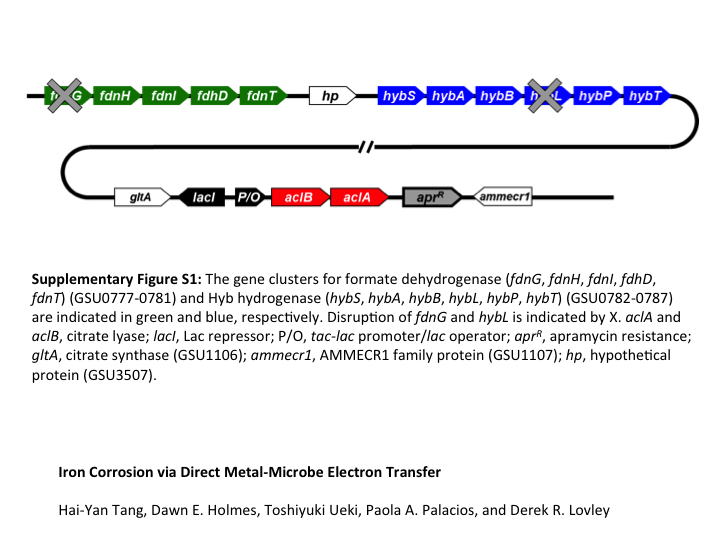

Supplement: FIG S1 [file mBio.00303-19-sf001.tif]
