# Supplementary material for: Iron Corrosion via Direct Metal-Microbe Electron Transfer
Source: mBio. 2019 May 14;10(3):e00303-19. doi: 10.1128/mBio.00303-19 (PMC6520446; doi:10.1128/mBio.00303-19)
Supplement: TABLE S2 [file mBio.00303-19-st002.docx]

**Supplementary Table S2:** Primers used to construct various deletion mutants and complement strains.

| **Primer Name** | **Propose** | **Primer sequence (5’-3’)** |
| --- | --- | --- |
| omcS up-f | omcS mutant construction | GCCTGGGCAACTACTACATCTC |
| omcS up-r |  | TATCCTAGGCGTTATCGTGAACATAGC |
| omcS down-f |  | TATCCTAGGGCGGTGCAACATC |
| omcS down-r |  | GAGAGGGCCATCTGGAATCG |
| omcS comp-f | omcS mutant complementation | ATGGATCCTCAAGGTAACGCACGACTGA |
| omcS comp-r |  | GTCTCGAGAGAGTCTTGAAGCGTTTCAT |
| omcZ up-f | omcZ mutant construction | CTCGTCTGTCGCTGCTAGTT |
| omcZ up-r |  | TATCCTAGGTCATTCCTTTCTGCT |
| omcZ down-f |  | TATCCTAGGCAGCCCGGACTT |
| omcZ down-r |  | GCCGGTTCAATGGACGAA |
| omcZ comp-f | omcZ mutant complementation | GCATATGAATTCAATTCAAGAAAGGAGCAGAAAGGAATG |
| omcZ comp-r |  | GCATCAAAGCTTTACCGTTTGACTTTCTTCGGAGC |
| gentAvr-f | Gentamycin cassette | TCCCTAGGTCGAATTGACATAAGCCTGTTC |
| gentAvr-r |  | TCCCTAGGGAACGAATTGTTAGGTGGCGGTA |
